# HPV‐Related Pelvic Squamous Cell Carcinoma of Unknown Primary: Two Case Studies

**DOI:** 10.1002/cnr2.70402

**Published:** 2025-12-03

**Authors:** Sepideh Soltani, Sahar Dashti, Maryam Garousi, Elahe Mirzaee, Majid Kaheh, Masoome Zolfaghari, Maryam Abolhasani, Alireza Nikoofar

**Affiliations:** ^1^ Department Of Radiation Oncology, School of Medicine Iran University of Medical Sciences Tehran Iran; ^2^ Department of Internal Medicine, School of Medicine Alborz University of Medical Sciences Karaj Iran; ^3^ Department of Radiology, School of Medicine Iran University of Medical Sciences Tehran Iran; ^4^ Oncopathology Research Center Iran University of Medical Sciences IUMS Tehran Iran; ^5^ Department of Pathology, Hasheminejad Kidney Center, School of Medicine Iran University of Medical Sciences Tehran Iran

**Keywords:** cancer of unknown primary, HPV, pelvic cavity, squamous cell carcinoma

## Abstract

**Background:**

Cancer of unknown primary (CUP) presents diagnostic and management challenges, particularly when associated with rare subsets such as pelvic squamous cell carcinoma (SCC) of unknown primary origin. Human papillomavirus (HPV) is increasingly recognized as a prognostic and potentially predictive biomarker. HPV‐associated SCCs often demonstrate better response to treatment and improved outcomes.

**Cases:**

We present two cases of pelvic SCC with unknown primary origin, both positive for HPV genotype 16. Case 1 involved a 54‐year‐old woman with persistent abdominal pain who was diagnosed with an infiltrative 110 × 100 × 65 mm tumoral mass on the right side of the pelvic cavity, significantly involving the right iliac bone and right iliopsoas muscle; despite chemotherapy, the patient developed metastases. Case 2 featured a 46‐year‐old woman with progressive left lower limb pain, whose pelvic SCC was incidentally discovered on imaging with an 80 × 75 mm mass with an abnormal signal in the left iliac bone with extension to the left iliopsoas muscle involving the lower aspect of the iliopsoas muscle, and also involving the anterior aspect of the left sacral bone. She achieved a complete response to chemotherapy and chemoradiotherapy, with no evidence of recurrence during follow‐up.

**Conclusion:**

HPV‐associated pelvic SCC of unknown primary presents both diagnostic complexity and therapeutic opportunity. The detection of HPV genotype 16 in both cases supports a growing body of case‐based evidence suggesting a potential association with a favorable prognosis. However, further studies are needed to clarify its role in guiding management.

## 1 Introduction

Patients diagnosed with cancer of unknown primary (CUP) have metastatic cancer that has spread to other parts of the body, but the primary tumor cannot be identified [1]. CUP is a rare condition, accounting for 3% to 5% of all invasive cancers [2]. It is the seventh to eighth most common malignancy and the fourth most common cause of cancer deaths. Diagnosing and managing CUP is challenging, but recent advancements in specific pathology investigations, serum tumor biomarkers, modern imaging technology, and new treatment modalities have improved patient outcomes [3, 4, 5].

Squamous cell carcinomas (SCCs) make up only about 5% to 10% of cancer cases with unknown origins [6]. While commonly found in the head and neck, SCCs can also arise in other anatomical regions [7]. Among these, HPV‐positive pelvic or retroperitoneal SCCs of unknown primary origin are exceptionally rare and have only been reported in a limited number of case studies [8, 9, 10].

HPV is frequently implicated in cervical, anal, and vulvar cancers. Its prognostic relevance is well established in oropharyngeal SCC, where p16 positivity serves as a surrogate marker; however, its clinical utility in other anatomical sites remains less clearly defined [11, 12, 13]. Its presence has been linked to distinct tumor biology, improved treatment response, and better prognosis in other anatomical sites, making HPV status a potentially valuable factor in guiding clinical management [14, 15]. Patient risk factors, clinical presentation, imaging results, and HPV status are pivotal factors in determining treatment approaches [6].

This manuscript discusses two cases of HPV‐positive pelvic SCC of unknown primary, a diagnosis that necessitates ruling out all potential primary sites, including the genitourinary tract and anorectal areas. Given its rarity, our discussion builds upon previous case reports to enhance the understanding of its clinical presentation and management [16].

Our aim is to offer a clinical perspective on these rare cases, contribute to the existing literature on HPV‐related malignancies, and add to the limited data available on this topic.

## 2 Case Reports

### 2.1 Case 1

#### 2.1.1 Case Presentation

**FIGURE 1:**
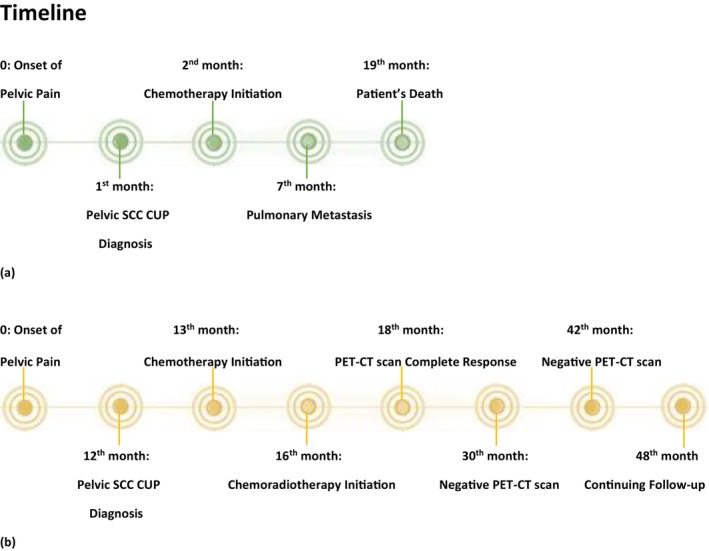
(a) Case 1 timeline, (b) Case 2 timeline, CUP, cancer of unknown primary; PET‐CT scan, Positron Emission Tomography and Computed Tomography; SCC, squamous cell carcinoma.

A 54‐year‐old woman with a known case of controlled diabetes mellitus and heart failure presented to the Pars Hospital clinic in February 2018 with a complaint of persistent abdominal pain for about 1 month (see case presentation timeline, Figure 1a).

She had no significant medical history related to gynecological or gastrointestinal abnormalities. Concerned about the potential underlying causes, her physician conducted a thorough physical examination and ordered various diagnostic tests, including blood work and imaging studies.

#### 2.1.2 Clinical Investigations and Diagnosis

Blood tests revealed the following: CBC values were hemoglobin at 12.8 g/dL, hematocrit at 38%, white blood cell (WBC) count at 6.2 × 10^3^/μL, and platelet count at 240 × 10^3^/μL. C‐reactive protein (CRP) was elevated at 8 mg/L. Tumor marker levels were CA‐125 at 20 U/mL, CEA at 0.2 ng/mL, and CA‐19‐9 at 6.43 U/mL, all of which were within normal limits.

### 2.2 Image Findings

**FIGURE 2:**
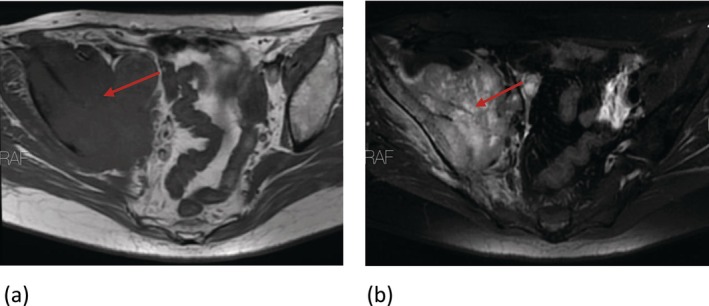
(a) An isoT1‐weighted image (T1WI), (b) high T2‐weighted image (T2WI) _ Short tau inversion recovery (STIR) sequence shows an infiltrative tumoral mass on the right side of the pelvic cavity, significantly involving the right iliac bone and right iliopsoas muscle, with the area where the mass is in close proximity to and displacing the adjacent fat planes (red arrow).

Her sonography showed a solid mass in her pelvic cavity. The Magnetic Resonance Imaging (MRI) showed an abnormal mass size of about 110 × 100 × 65 mm on the right side of the pelvic cavity involving adjacent structures (Figure 2). The anus, uterus, and bladder appeared normal.

The fluorodeoxyglucose‐positron emission tomography (FDG‐PET) CT scan showed a lytic destructive bony lesion in the right iliac bone with increased metabolic activity (SUV_max_ = 11.9, Size: 100 mm). There were no other areas of concern, confirming a diagnosis of pelvic SCC with an unknown primary origin.

### 2.3 Biopsy and Immunohistochemistry

**FIGURE 3:**
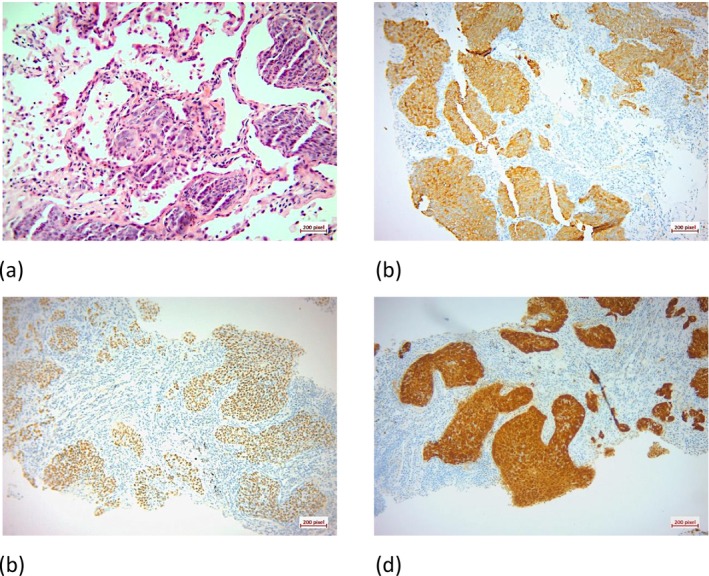
(a) H&E staining shows nests of tumoral cells with enlarged nuclei and eosinophilic cytoplasm (×20), (b) Positive immunoreaction for CK 5/6 in tumoral cells (×10). (c) Positive immunoreaction for p63 in tumoral cells (×10). (d) Positive immunoreaction for p16 in tumoral cells (×10).

CT‐guided core needle biopsy of the pelvic mass revealed poorly differentiated carcinoma. Additional immunohistochemistry (IHC) markers indicated a squamous origin, and positive staining for p16 suggested an HPV association (Figure 3).

Further evaluations, including a pap smear, urine cytology, and proctoscopy, showed no abnormalities.

#### 2.3.1 Treatment and Management

According to ESMO guidelines for CUP and squamous cell carcinomas, systemic chemotherapy is recommended in cases where surgical resection is not feasible [17]. Due to her medical comorbidities and surgical evaluation, she was deemed inoperable, and the decision was made to approach first by chemotherapy and, after response evaluation, chemoradiotherapy.

She received carboplatin at the target area under the curve 6 mg/mL/min followed by paclitaxel 200 mg/m^2^ as a 3‐h infusion and granulocyte colony‐stimulating factor from days 5 to 12, repeated every 3 weeks [18]. Her pain was partially relieved by the sixth course; her restaging PET CT scan showed a lytic lesion in the right iliac bone, with mild metabolic activity (SUV_max_ = 1.4) and a maximum diameter of 20 mm, but in her right lung, a hypermetabolic nodule containing small lucency in the right upper lobe was detected (SUV_max_ = 6.5) with a maximum diameter of 25 mm. A core needle biopsy of the lung nodule indicated poorly differentiated carcinoma with strong P16 staining and IHCs compatible with her pelvic SCC primary.

Radiation therapy was considered after chemotherapy; however, due to the patient's medical comorbidities (including heart failure), overall frailty, and the emergence of distant metastases, she was no longer a candidate for local radiotherapy.

Immunotherapy was not pursued given her tumor's PD‐L1 TPS score of < 1% and her personal decision to decline further systemic treatment despite medical recommendations.

#### 2.3.2 Outcome

She died 1 year after her treatment completion due to pulmonary complications.

#### 2.3.3 Case Summary

The patient with pelvic SCC of unknown primary underwent chemotherapy with carboplatin and paclitaxel, followed by chemoradiotherapy due to inoperability. A partial response in the pelvic mass was observed, but new lung metastasis was identified on restaging. Despite the improvement in the pelvic lesion, the development of lung metastasis, absence of PD‐L1 expression, and refusal of further treatment led to disease progression and eventual death from pulmonary complications.

### 2.4 Case‐2

#### 2.4.1 Case Presentation

A 46‐year‐old woman with no previous medical or drug history presented with a new onset, persistent deep pain in her left lower limb, described as non‐radiating, constant in nature, and refractory to analgesics. Initial history, physical examination, and workups showed no abnormalities, and despite conservative management for a year, her condition did not improve (see case presentation timeline, Figure 1b).

#### 2.4.2 Clinical Investigations and Diagnosis

At baseline, the lab results showed a white blood cell count of 6.2 × 10^3^/μL, hemoglobin of 12.5 g/dL, and platelet count of 220 × 10^3^/μL. Liver function tests were within normal limits, with ALT at 30 U/L and AST at 28 U/L. Renal function was normal, with a serum creatinine of 0.8 mg/dL and BUN of 12 mg/dL. Lactate dehydrogenase (LDH) was mildly elevated at 210 U/L, while tumor markers like CEA were 1.2 ng/mL, CA‐125 was 18 U/mL, and CA‐19‐9 was 22 U/mL, all within normal limits.

### 2.5 Imaging and Biopsy Results

**FIGURE 4:**
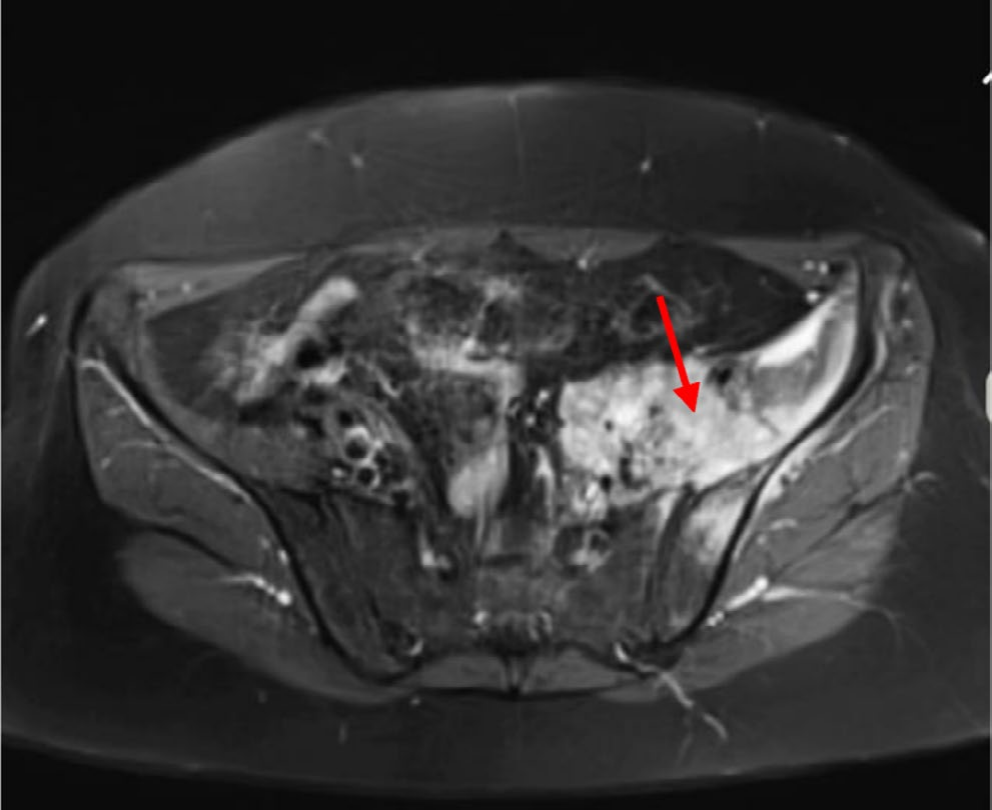
T1_weighted image with fat suppression shows an 80 × 75 mm mass with an abnormal signal in the left iliac bone with extension to the left iliopsoas muscle involving the lower aspect of the iliopsoas muscle, causing enlargement of this muscle and also involving the anterior aspect of the left sacral bone. There is obstruction of the left ureter by this mass at the region of the entrance to the pelvis, causing dilatation of the left ureter (red arrow).

Her primary physician conducted a pelvic spine MRI, which incidentally revealed a soft tissue mass lesion on the left side of the pelvic cavity (Figure 4). As a result, she was referred to the Pars Hospital clinic in November 2020 for further evaluation.

**FIGURE 5:**
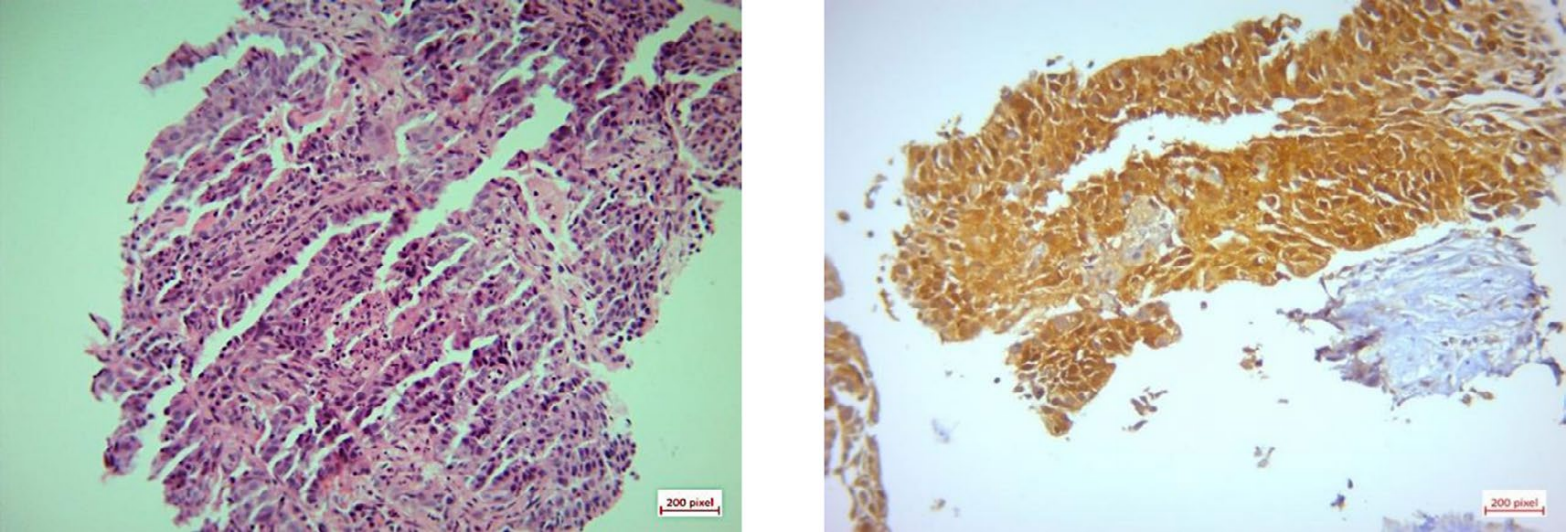
(a) H&E staining shows nests of tumoral cells with enlarged nuclei and eosinophilic cytoplasm (x20), (b) Positive immunoreaction for P 16 in tumoral cells (×40).

Subsequently, a core needle biopsy was performed, which indicated a high‐grade carcinoma with features suggestive of squamous cell carcinoma (Figure 5).

Further evaluations of the cervix, anus, renal pelvis, skin, and other organs yielded no sources of cancer.

**FIGURE 6:**
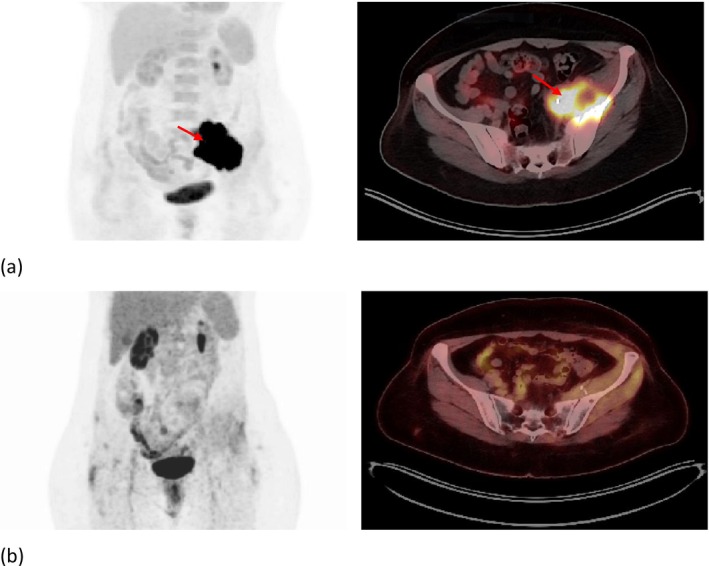
(a) pre‐treatment PET‐CT scan showing A soft tissue mass measuring 75 × 65 mm with SUV_max_ of 32.2 and central hypoactivity in the left hemipelvis region involving the left psoas, left iliac (and apparently left gluteus medius) muscles, and the left iliac bone wing (red arrow), (b) post‐treatment PET CT scan showing complete response, with no residual mass and no apparant abnormal FDG uptake.

To gather additional information, a PET‐CT scan was performed. It revealed a soft tissue mass with an intense FDG uptake of 32.2 and a maximum diameter of 75 mm on the left side of the pelvis and no abnormal metabolic activity in the rest (Figure 6a).

#### 2.5.1 Treatment and Management

With the impression of pelvic SCC of unknown primary, the patient underwent treatment consisting of chemotherapy with gemcitabine 1250 mg/m^2^ on days 1 and 8 and cisplatin 100 mg/m^2^ IV on day 1, repeated every 3 weeks for three courses [19]. This regimen was selected based on institutional practice and prior reports indicating tolerability and efficacy in non‐head and neck SCCs, particularly where HPV association was suspected, and standard CUP regimens were not clearly defined.

Chemotherapy was followed by 5000 cGy radiation therapy administered in 25 fractions with 3D conformal technique, along with weekly cisplatin 40 mg/m^2^ concomitantly the radiation field was confined to the gross tumor volume (GTV) without elective nodal coverage in accordance with institutional practice and due to the absence of nodal involvement on imaging. After treatment evaluation, her PET CT scan showed no residual mass, indicating a complete response (Figure 6b).

#### 2.5.2 Outcome

Over the course of the past 3 years, regular 3‐month intervals of physical examinations and annual PET‐CT scan follow‐ups revealed no indications of tumor‐related abnormalities, providing positive reassurance about her current condition.

#### 2.5.3 Case Summary

The patient diagnosed with pelvic SCC of unknown primary received a combination of chemotherapy with gemcitabine and cisplatin, followed by chemoradiotherapy using cisplatin. After completing three cycles of chemotherapy and radiation therapy, the patient achieved a complete response, as evidenced by the post‐treatment PET‐CT scan showing no residual tumor. Subsequent follow‐up examinations, including annual PET‐CT scans, have demonstrated no signs of recurrence, with the patient remaining disease‐free over the past 3 years.

Recently, the DNA of the cases' paraffin‐embedded blocks from the core needle biopsy of their primary site, pelvic mass, was extracted using the Cedbio kit (Iran). Conventional PCR was performed using an ABI Thermal Cycler (USA), and HPV testing was performed using a reverse dot blot hybridization assay (Master Diagnostica, Spain). Both cases were positive for HPV genotype 16 (Table 1).

**TABLE 1:** Summary of clinical features, treatments, and outcomes in pelvic SCC Cases.

Feature	Case 1: 54‐year‐old woman	Case 2: 46‐year‐old woman
Presenting complaint	Persistent abdominal pain for 1 month	Progressive persistent left lower limb pain
Medical history	Controlled diabetes mellitus, heart failure	No previous medical or drug history
Tumor markers	CA‐125: 20 U/mL, CEA: 0.2 ng/mL, CA‐19‐9: 6.43 U/mL (all normal)	CA‐125: 18 U/mL, CEA: 1.2 ng/mL, CA‐19‐9: 22 U/mL (all normal)
Imaging results	Solid mass: 110 × 100 × 65 mm	Soft tissue mass: 80 × 75 mm
Histopathological diagnosis	Poorly differentiated carcinoma, squamous origin	High‐grade carcinoma, squamous origin
Immunohistochemistry	Positive: CK 5/6, p63, p16	Positive: P16
HPV testing result	HPV Genotype 16 positive	HPV Genotype 16 positive
Treatment regimen	Chemotherapy: Carboplatin, Paclitaxel; Chemoradiotherapy	Chemotherapy: Gemcitabine, Cisplatin; Chemoradiotherapy (Cisplatin)
Response to treatment	Partial response in pelvic mass, new lung metastasis	Complete response, no residual mas
Outcome	Died 1 year post‐treatment due to pulmonary complications	Disease‐free for 3 years post‐treatment

## 3 Discussion

### 3.1 Introduction to Pelvic Squamous Cell Carcinoma of Unknown Primary Origin

The cases presented in this report offer observations on a rare and clinically challenging subset of cancer, pelvic squamous cell carcinoma (SCC) of unknown primary origin, noteworthy for its association with human papillomavirus (HPV) positivity. Recognizing the significance of these cases is essential for understanding their natural history, as well as diagnostic and therapeutic implications.

### 3.2 Cancer of Unknown Primary (CUP)

CUP refers to metastatic tumors where the primary site remains unidentified despite thorough evaluation. It is characterized by early dissemination, aggressive behavior, and unpredictable metastatic patterns [20]. Pelvic SCC of unknown origin is particularly rare and has been reported mainly through case studies, often presenting with vague symptoms like abdominal or pelvic pain, particularly in women [21].

### 3.3 Molecular Biology and Hypotheses

Based on the patients' medical history and examination, along with the pathologic and molecular features, the possible causes of squamous cell CUP in the pelvis can be hypothesized [6, 9, 22]. Efforts have been made to understand its molecular biology, but gene expression profiling has not yet identified any signature specific to CUP [23].

### 3.4 The Role of HPV and p16 Positivity

HPV‐related SCC is best known in cervical, anal, and oropharyngeal cancers, where HPV positivity—often detected by p16 overexpression—has been linked to a favorable treatment response and prognosis.

Although less common in pelvic CUP, HPV detection can help guide treatment and identify mucosal sites at risk [8, 9, 24].

p16 is widely used as a surrogate marker for transcriptionally active HPV, particularly in oropharyngeal SCC. However, as Doll et al. show, its correlation with HPV DNA is weaker in non‐oropharyngeal sites like oral SCC, highlighting its limitations outside those contexts [25].

Combining HPV DNA/RNA testing with p16 IHC improves diagnostic accuracy and may support de‐escalation strategies, especially in HPV‐driven cancers [24, 26].

### 3.5 Treatment Options

The optimal treatment for pelvic retroperitoneal squamous cell CUP remains uncertain. Both surgery and radiation are viable options, particularly for HPV‐induced cancers, suggesting potential sensitivity to radiation therapy. Surgery or chemoradiation shows promise in achieving favorable outcomes, although consensus is lacking due to its rarity. In the era of targeted therapies, precise histopathological and molecular classification is essential for tailoring the most effective treatment strategy for individual patients [24, 27].

Emerging evidence suggests that immunotherapy may benefit HPV‐positive SCC‐CUP, especially after progression on standard treatments. A case by Komura et al. reported a favorable response to nivolumab in a p16‐positive pelvic SCC‐CUP patient, supporting its potential as a treatment option [28].

Also due to recent advancements in therapeutic options, there are vaccines that offer promising strategies for directly targeting HPV in HPV‐positive cancers, potentially leading to long‐term immunity and the prevention of cancer recurrence, which could inform future treatment approaches for pelvic squamous cell carcinoma of unknown primary origin [29].

### 3.6 Recurrence and Follow‐Up Care

Furthermore, according to reports, these lesions may act as precursors to malignancies arising from them, with the possibility of recurrence in the future, sometimes years later. This highlights the imperative for extended and more vigilant follow‐up care for these patients [30].

### 3.7 Case Descriptions and Patient Outcomes

The presented cases involved two female patients, aged 47 and 54, one premenopausal and the other postmenopausal, both experiencing chronic pelvic pain. Their diagnoses revealed squamous cell carcinoma of unknown primary, with positivity for both P16 and HPV type 16. Despite the consideration of surgical resection in select cases, both patients were deemed inoperable due to the extent of the disease and associated medical comorbidities. However, their treatment outcomes diverged: while one patient under chemotherapy exhibited metastases, the other achieved a complete response to chemotherapy and subsequent chemoradiotherapy.

These findings are consistent with prior reports. Isbell et al. described three similar HPV‐positive pelvic SCC‐CUP cases, two of which responded well to chemoradiation [6]. Our Case 2 mirrors these favorable outcomes. In contrast, Case 1 aligns with Agrawal et al., where the disease initially regressed but later progressed [31].

A literature review by El Rassy et al. also supports a more favorable prognosis for HPV‐related pelvic SCC‐CUP, which is often responsive to chemoradiotherapy [32]. These studies support the notion that HPV positivity may identify a prognostically favorable subgroup amenable to curative‐intent treatment, consistent with the complete response observed in Case 2.

The contrasting outcomes of these two cases can be attributed to several key factors, including tumor burden, metastatic spread, treatment response, and overall physical condition. In Case 1, the tumor was larger with significant local invasion, leading to a poorer prognosis. Conversely, Case 2 had a smaller, localized tumor, which made it amenable to curative treatment [33]. Additionally, Case 1 exhibited metastatic spread on FDG‐PET, which worsened the prognosis, while Case 2 had no metastasis, resulting in a better prognosis, as confirmed by PET‐CT scan imaging [34]. Treatment response also played a significant role; Case 1 had a partial response to treatment and eventually progressed, which is typical in advanced‐stage disease. In contrast, Case 2 responded well to treatment, reaching a complete response [35]. Furthermore, the presence of comorbidities in Case 1 compromised overall health and treatment tolerance, leading to a less favorable outcome. In contrast, Case 2 had no such issues, allowing for better treatment adherence [36]. While these factors explain much of the difference in disease progression and treatment outcomes between the two patients, it is important to consider that molecular and genetic factors may also have influenced these results, requiring further investigation for a comprehensive understanding.

### 3.8 Clinical Insights and Contributions

These two cases of pelvic squamous cell carcinoma (SCC) of unknown primary origin contribute to the existing literature by highlighting the diverse clinical presentations and outcomes in patients with this rare condition. Case 1 demonstrates the progression of the disease despite chemotherapy and chemoradiotherapy, underscoring the challenges in treating inoperable pelvic SCC and the potential for distant metastasis, such as the development of lung metastasis. In contrast, Case 2 showcases a complete response to chemotherapy and radiation, offering a more optimistic outcome for patients with this rare diagnosis. Surgical resection was not pursued in Case 2 due to deep pelvic extension and proximity to critical neurovascular structures; after multidisciplinary review, definitive chemoradiation was selected to optimize local control while minimizing treatment‐related morbidity. Both cases are unique in that they underscore the importance of a thorough diagnostic workup and individualized treatment approaches. Additionally, the HPV genotype 16 positivity in both cases adds an intriguing molecular aspect that could provide insights into the potential viral etiology of pelvic SCC. However, the generalizability of these findings is limited due to the small sample size of just two cases, and further studies are needed to validate these observations and assess the efficacy of various treatment regimens.

## 4 Conclusion

To conclude, these cases of pelvic squamous cell carcinoma (SCC) with unknown primary origin, both positive for human papillomavirus (HPV), underscore the complexities in diagnosis and management. They prompt important questions regarding the role of HPV in prognosis and treatment decisions, as well as the potential impact of HPV vaccination. Emphasizing the need for targeted research into HPV's role in non‐cervical pelvic cancers, future studies should focus on the molecular mechanisms driving this association and explore potential therapeutic interventions. Collaborative efforts involving multi‐center studies, genomic analysis, and HPV‐specific therapeutic trials could significantly enhance our understanding and improve treatment strategies for these rare and challenging cancers.

Clinicians encountering similar cases should prioritize a thorough diagnostic workup, including HPV testing and p16 immunohistochemistry, as these may inform prognosis and guide treatment intensity. In cases where standard treatment guidelines are lacking, multidisciplinary discussions and individualized approaches are essential.

Further research is necessary, particularly focused on molecular characterization, HPV‐driven oncogenesis outside the cervix, and the development of targeted therapies. Such advancements could pave the way for more personalized and effective management of HPV‐related pelvic SCC of unknown primary.

## Author Contributions

**Sepideh Soltani:** writing – original draft (lead); writing – review and editing (lead). **Sahar Dashti:** visualization (equal); data curation (equal). **Maryam Garousi:** writing – review and editing (equal). **Elahe Mirzaee:** data curation (equal); writing – review and editing (equal). **Majid Kaheh:** data curation (equal); visualization (equal). **Masoome Zolfaghari:** visualization (equal); writing – review and editing (equal). **Maryam Abolhasani:** data curation (equal); writing – review and editing (equal). **Alireza Nikoofar:** conceptualization (lead); supervision (lead); validation (lead).

## Funding

The authors have nothing to report.

## Ethics Statement

We obtained written informed consent from both patients for the publication of their case details and any accompanying images. For the patient who is deceased, consent was obtained from a legally authorized representative. This case report was conducted in accordance with the ethical standards set by the institution. As the study involved a retrospective review of medical records and no direct patient intervention, it was not subjected to formal ethical review approval. Nonetheless, all patient information was anonymized to ensure confidentiality and protect privacy.

## Consent

Written informed consent was obtained from the patients for the publication of this case report and any accompanying images. Copies of the written consent are available for review by the Editor‐in‐Chief of this journal.

## Conflicts of Interest

The authors declare no conflicts of interest.

## Data Availability

The data that support the findings of this study are available on request from the corresponding author.
